# Size-selected growth of transparent well-aligned ZnO nanowire arrays

**DOI:** 10.1186/1556-276X-7-517

**Published:** 2012-09-21

**Authors:** Junsheng Yu, Zhaolin Yuan, Shijiao Han, Zhu Ma

**Affiliations:** 1 State Key Laboratory of Electronic Thin Films and Integrated Devices, School of Optoelectronic Information, University of Electronic Science and Technology of China (UESTC), Chengdu, 610054, People’s Republic of China; 2 School of Physics and Electronic Information Engineering, Shaanxi University of Technology, Hanzhong, 723000, People’s Republic of China

**Keywords:** ZnO nanowire arrays, Chemical bath deposition, Optical transmission, Hybrid solar cell

## Abstract

This paper reports the effect of precursor concentration, growth temperature, and growth time on the size and density of ZnO nanowire arrays (ZNAs). The well-aligned ZNAs were grown on indium tin oxide substrate using a facile chemical bath deposition method. The results showed that the ZnO nanowires could be tailored to the desired sizes with a simple variation of the growth parameters. Optical transmission spectra revealed a sufficient transparency of the ZNAs, qualifying them for photovoltaic and other optoelectronic applications. An inverted hybrid solar cell was fabricated using the ZNAs as the electron collecting layer, and the solar cell exhibited a power conversion efficiency of 0.91%.

## Background

As an important wide-bandgap semiconductor, ZnO possesses remarkable optical, electrical, and optoelectronic properties, thus being of immense research interest [1-4]. Recently, well-aligned ZnO nanowire or nanorod arrays have been extensively studied as a promising candidate for applications in electroluminescent devices [5,6], field emission devices [7], solar cells [8-14], nanogenerators [15-17], and chemical sensors [18-20].

To grow well-aligned ZnO nanowire arrays (ZNAs), various synthesis methods have been utilized, such as thermal evaporation [7], chemical vapor transport and condensation [18], and vapor–liquid-solid growth [20]; the complex process, sophisticated equipment, and high temperatures make it hard to use them on a wide range of substrates. In contrast, the chemical bath deposition method shows its great advantages due to its much easier operation, very low temperature (≤ 95°C) growth, high potential for scale-up, and low-cost. The facile and low-temperature growths favor the applications of well-aligned ZNAs as electroluminescent diodes and hybrid solar cells [8-10]. Previous reports [21,22] indicate that the sizes (diameter and length) of ZnO nanowires or nanorods play an important role on the performance of building ZNA hybrid solar cells. In light of our experimental observations, several factors, including concentration of precursors, growth temperature, and growth time, had great effect on the sizes and density of well-aligned ZNAs grown via the chemical bath deposition method. However, up to now, only a few works on size-controlled and transparent well-aligned ZNAs are available [21,23].

In this work, we present size-controlled and transparent ZNAs by a facile chemical bath deposition method. It is shown that the nanowires can be tailored to the desired sizes with a simple variation of the growth parameters. The optical transmission of the obtained well-aligned ZNAs in the visible wavelength region was also discussed. Exemplarily, a hybrid solar cell was constructed and fabricated, consisting of the as-prepared ZNAs and polymer hybrid bulk heterojunction architecture.

## Methods

### Materials and substrates

Regioregular poly(3-hexylthiophene-2,5-diyl) (P3HT, 99.5%) and (6,6)-phenyl-C_61_-butyric acid methyl ester (PCBM, 99.5%) were purchased from Lumtec (Hsin-Chu, Taiwan) and used as received. Other chemicals (Kelong Chemical Agent, Chengdu, China) used in our experiments were of analytical reagent grade without further purification. All the aqueous solutions were prepared using distilled water (resistivity = 18.2 MΩ cm). The indium tin oxide (ITO)-coated glasses (10 Ω/sq) were used as the substrate and first cleaned by ultrasonic agitation in detergent, then deionized water, acetone, and ethanol, successively. The cleaned substrates were then blown dry using nitrogen gas and treated with O_2_ plasma for 5 min.

### Preparation of ZNAs

ZNAs were prepared by the following two-step process, and the growth of the ZNAs was conducted following our previous work [24,25]. Briefly, zinc acetate dehydrate (Zn(CH_3_COO)_2_·2H_2_O) was dissolved in ethanol with a concentration of 5 mM. A droplet of the solution was coated onto treated ITO substrates, rinsed with clean ethanol after 10 s, and then blown dry with a stream of nitrogen gas. This coating step was repeated several times. The coated substrates were dried at room temperature and then annealed at 350°C for 20 min in air to yield the multilayers of ZnO seed. The zinc acetate deposition and decomposition procedure were carried out twice to ensure a complete and uniform coverage of ZnO seeds.

Then, the ZNAs were grown in 200 mL of equimolar aqueous solution of Zn(NO_3_)_2_·6H_2_O and hexamethylenetetramine (C_6_H_12_N_4_) in a conventional reaction flask. The ZnO seed layer-coated ITO substrate was immersed into the aqueous solution upside-down in the flask; the pH value of the growth solution was approximately 6.0 by adding aqueous ammonia. Various growth parameters such as concentration of precursors, growth temperature, and growth time were used in our experiments. Table 1 summarizes major experiments carried out in this work. After growth, the substrates were removed from the solution, rinsed with deionized water, and dried at 50°C in air.

**Table 1:** Growth parameters of ZnO nanowire arrays

**Sample**	**Growth temperature (°C)**	**Growth time (h)**	**Concentration of precursors (M)**
Sample A	73	2	0.025
Sample B	83	2	0.025
Sample C	93	2	0.025
Sample D	93	1	0.025
Sample E	93	3	0.025
Sample F	93	4	0.025
Sample G	93	2	0.01
Sample H	93	2	0.05
Sample I	93	2	0.1

### Preparation of ZNA/polymer hybrid solar cell

A hybrid solar cell was fabricated using the following procedure. The organic layers consisted of P3HT:PCBM (1:1 by weight ratio) were spin-coated on the top of sample C from a chlorobenzene solution with a concentration of 30 mg/ml in air, then annealed at 110°C for 15 min under vacuum (≤133 Pa). The 3-nm-thick MoO_3_ was deposited on the P3HT:PCBM layers under a pressure of 3 × 10^−3^ Pa as a buffer layer [24], followed by the deposition of 200-nm-thick Ag electrodes through a shadow mask.

### Characterization

The morphology of as-grown ZNAs was analyzed using a field-emission scanning electron microscope (FESEM, Hitachi S-4800, Chiyoda-ku, Japan); X-ray diffractometry (XRD) data were obtained on a Philips X'pert Pro MPD diffractometer (CuK_α_ radiation, *λ* = 1.54056 Å, Amsterdam, The Netherlands). Optical transmission was measured by a UV–vis spectrophotometer (SHIMADZU UV1700, Kyoto, Japan). The current–voltage characteristics of as-prepared solar cells in the dark and under illumination were recorded with a Keithley 4200 programmable voltage–current source (Cleveland, OH, USA) [26-28]. A solar simulator equipped with a xenon lamp with an illumination power of 500 W (CHF-XM35, Beijing Trusttech Co. Ltd., Beijing, China) was used as the light source. All measurements were performed under ambient conditions without device encapsulation.

## Results and discussion

**Figure 1:**
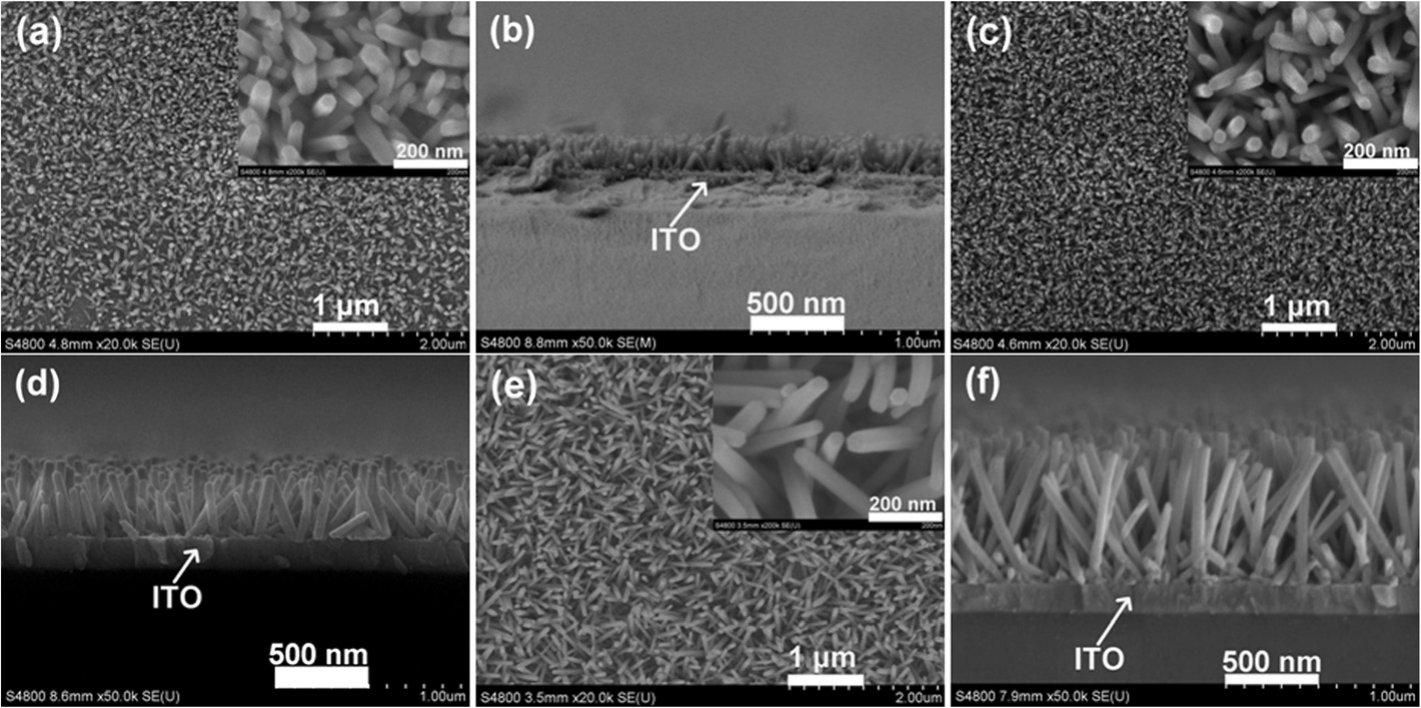
**FESEM images of samples (a, b) A, (c, d) B, and (e, f) C.** (**a**), (**c**), and (**e**) correspond to top views. (**b**), (**d**), and (**f**) correspond to cross-sectional views. The insets show high-magnification FESEM images.

In this study, we found that the different growth temperatures strongly influenced the nanowire length and density of ZNAs. Figure 1 shows the top-view, high-magnification (shown in the insets), and cross-sectional FESEM images of samples A, B, and C. There are only wirelike features with a hexagonal cross section for all samples, which are aligned in a dense array approximately perpendicular to the substrate surface. The ZNAs uniformly covered the entire substrate surface based on the FESEM result. The mean values of the nanowire dimension and array density for samples A, B, and C were estimated from a statistical evaluation of FESEM images and are summarized in Table 2. We note that with the enhancement of growth temperature, both the average length and array density obviously increase, and the average diameter of nanowires almost displays no change.

**Table 2:** Mean values of the nanowire diameter, length, and array density of the samples

**Sample**	**Diameter (nm)**	**Length (nm)**	**Density (×10**^**9**^**wires/cm**^**2**^**)**
Sample A	30	190	11.0
Sample B	35	340	18.0
Sample C	35	750	19.2
Sample D	20	200	18.0
Sample E	45	1,000	19.8
Sample F	50	1,000	20.4
Sample G	30	500	10.4
Sample H	40	850	21.6
Sample I	200	950	4.8

**Figure 2:**
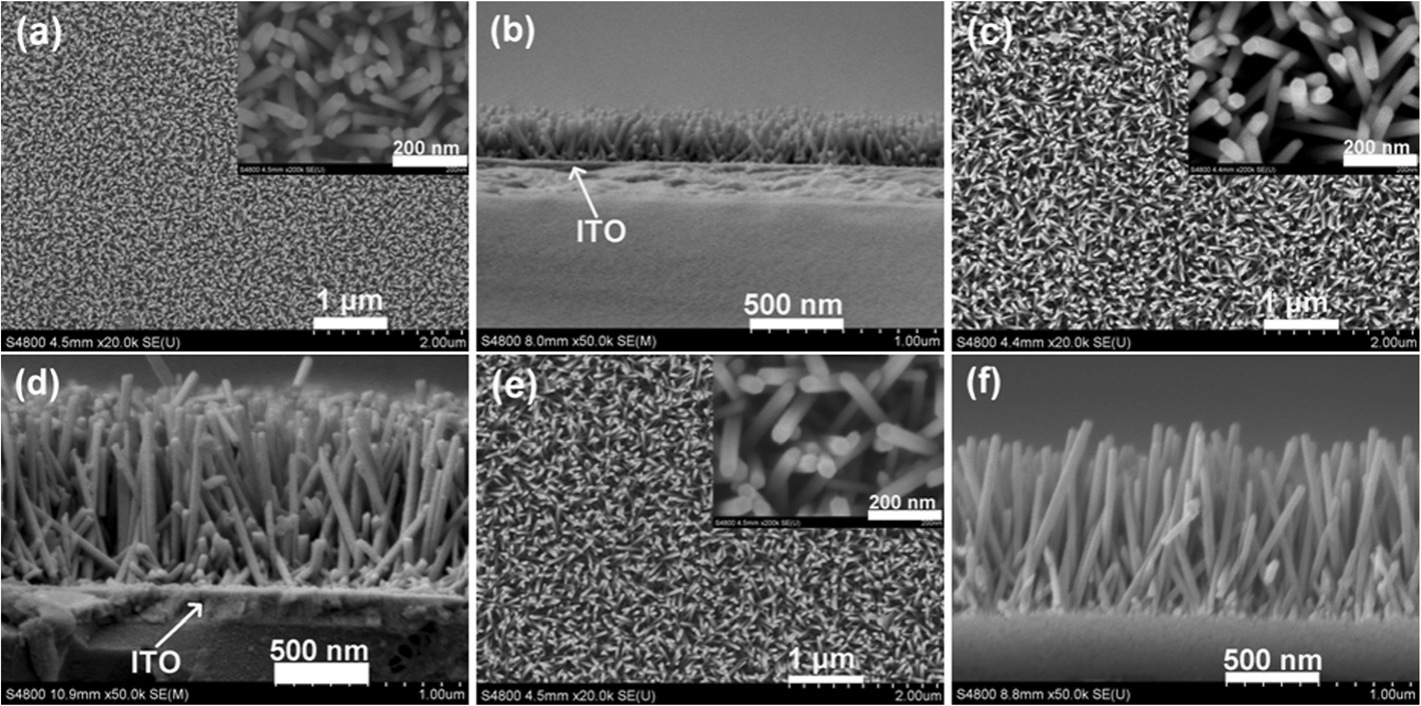
**FESEM images of samples (a, b) D, (c, d) E, and (e, f) F.** (**a**), (**c**), and (**e**) correspond to top views. (**b**), (**d**), and (**f**) correspond to cross-sectional views. The insets show high-magnification SEM images.

The effect of the growth time on the morphology and density of ZNAs was also investigated, as shown in Figure 2. It can be seen that the highly uniform and densely packed arrays of ZnO nanowires are successfully formed for samples D, E, and F. The mean values of the nanowire dimension and array density for samples D, E, and F are also summarized in Table 2. Comparing the average length, diameter, and array density among samples D, C, E and F, respectively, it can be seen that both the average diameter and array density slowly increase with elongating growth time. The average length rapidly increases as the growth time elongates from 1 to 3 h. However, further elongating the growth time, the average length shows no change.

**Figure 3:**
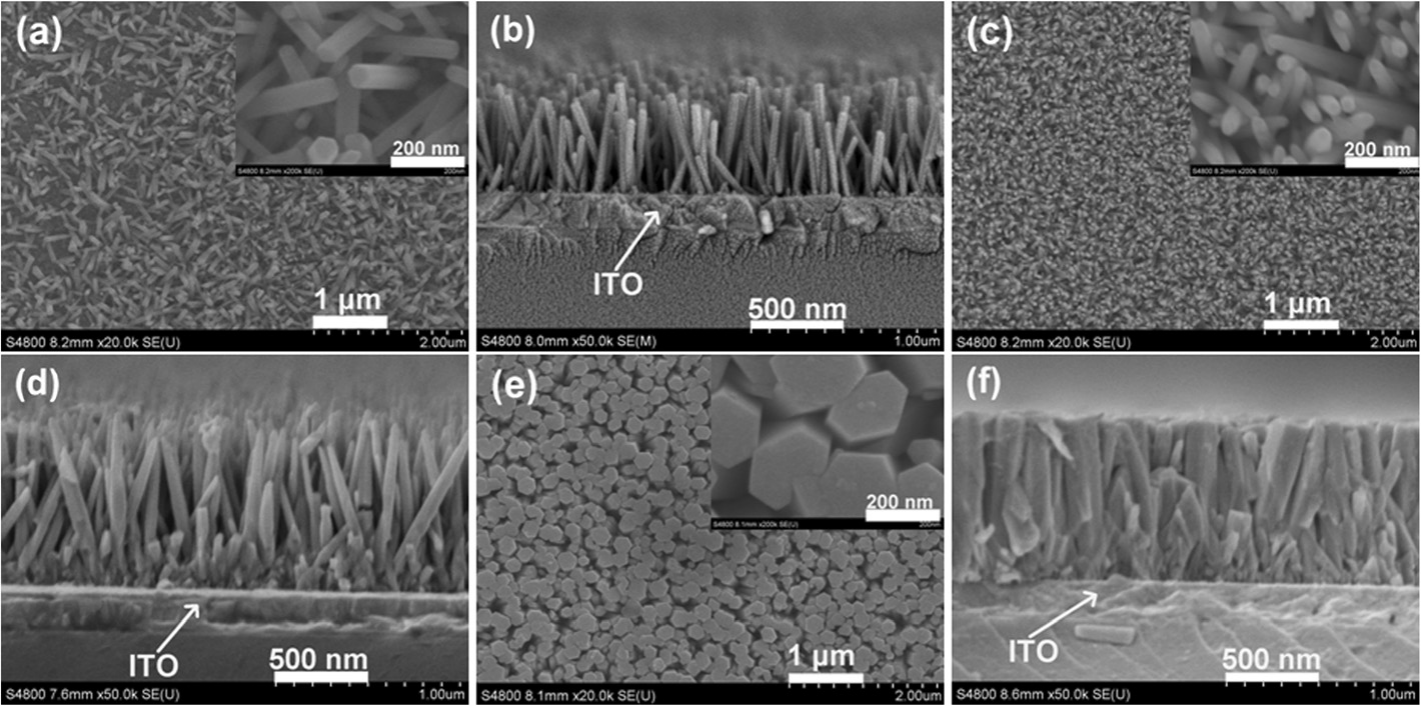
**FESEM images of samples (a, b) G, (c, d) H, and (e, f) I.** (**a**), (**c**), and (**e**) correspond to top views. (**b**), (**d**), and (**f**) correspond to cross-sectional views. The insets show high-magnification SEM images.

Figure 3 shows top-view, high-magnification (shown in the insets), and cross-sectional FESEM images of samples G, H, and I. The results reveal that well-aligned ZNAs are also obviously formed for samples G, H, and I. Also, the mean values of the nanowire dimension and array density for samples G, H, and I are summarized in Table 2. It can be seen that both the average diameter and length of the nanowires increase with the increased concentration of precursors from 0.01 to 0.1 M, as seen in samples G, C, H, and I in Table 2. The array density rapidly increases as the concentration of precursors ranged from 0.01 to 0.05 M. However, further increasing the concentration of precursors up to 0.1 M, the array density of ZNAs sharply decreases, which is attributed to the average diameter of the nanowires that becomes too large.

**Figure 4:**
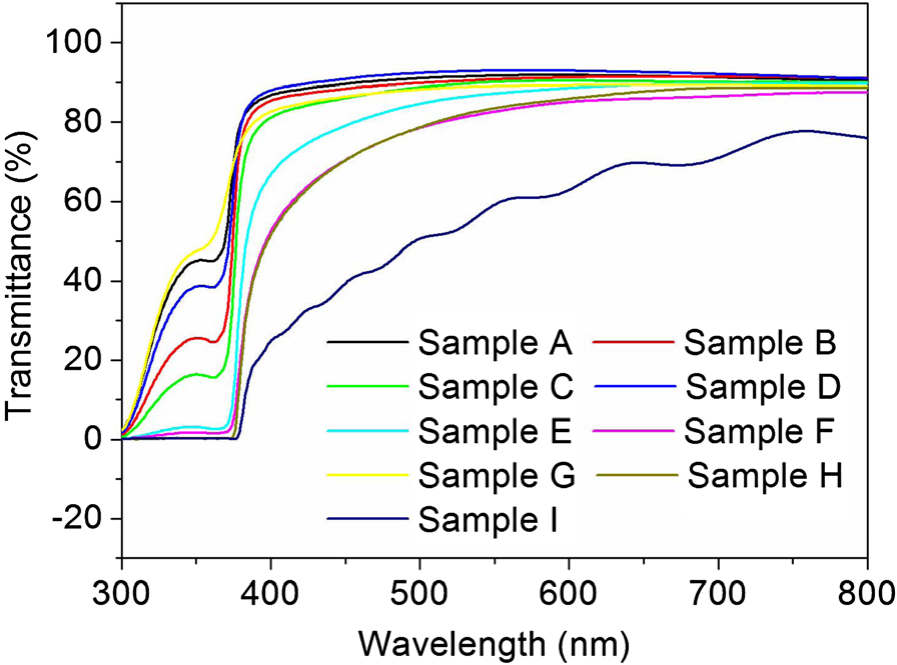
Optical transmittance spectra of samples A to I.

The optical transparency of all samples fabricated on ITO substrates was investigated. Figure 4 shows the optical transmittance spectra of samples A to I. With the exception of sample I, all of the samples show good optical transparency in the visible spectral range, with a transmittance above 80%. However, sample I displays a lower transmittance over a broad range of wavelengths as compared to other samples. This was due to the increase in light scattering from the large diameter of ZnO nanowires in sample I, which is consistent with the FESEM results.

**Figure 5:**
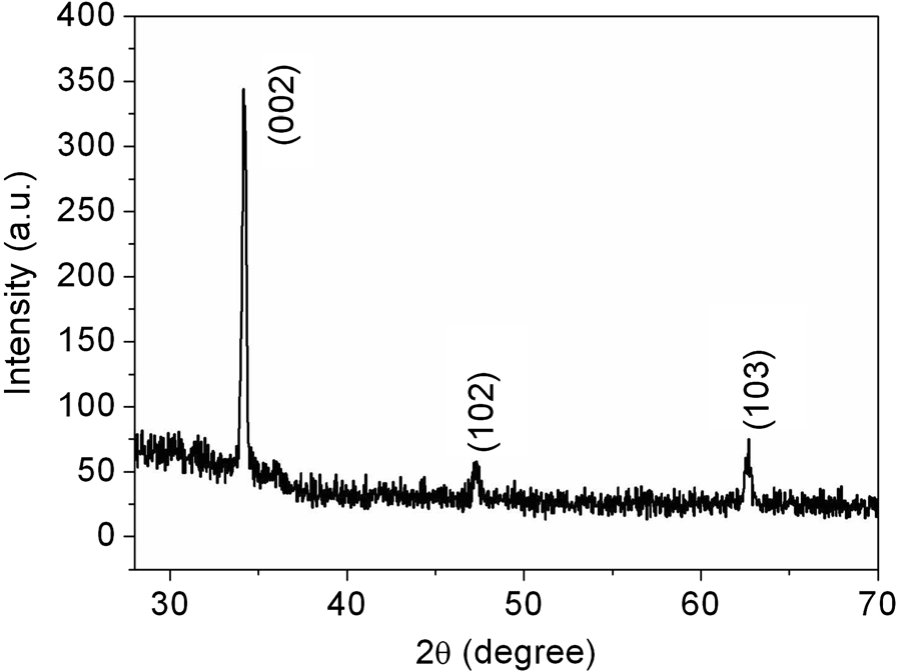
XRD pattern of sample C.

Figure 5 shows the XRD patterns of sample C. The XRD analysis reveals that all of the diffraction peaks of ZNAs can be indexed as those from the known wurtzite-structured (hexagonal) ZnO (JCPDS card no. 36–1451) and the (002) diffraction peak exhibits a substantially greater intensity. The result further demonstrates that as-grown ZNAs are much better aligned on the substrate and the nanowires exhibit preferred growth along the (0001) direction.

**Figure 6:**
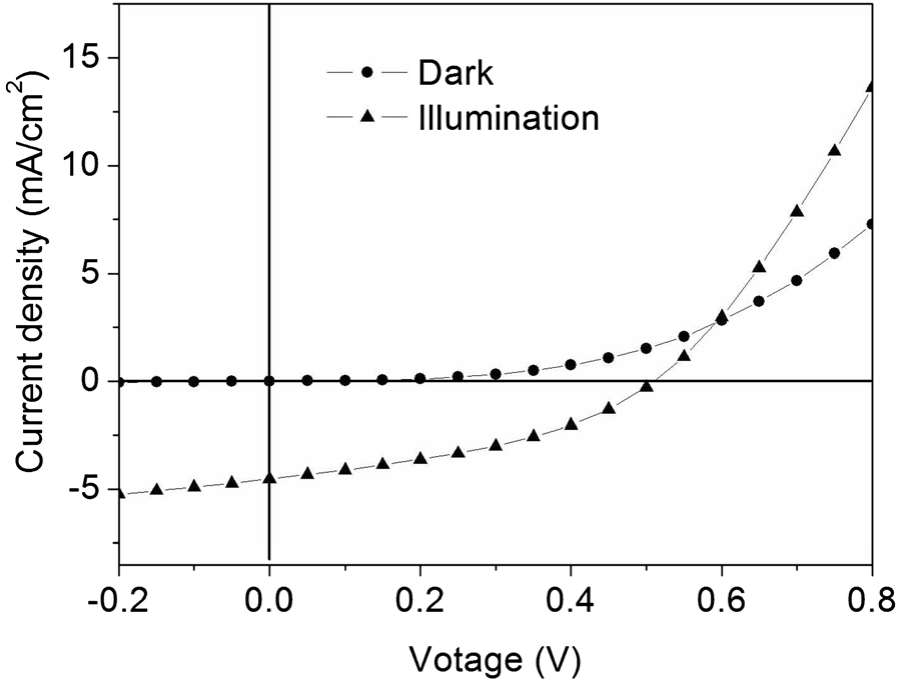
***J*-*V *characteristics of sample C/P3HT:PCBM/MoO**_**3**_**/Ag solar cell in the dark and under AM 1.5 illumination.** The light intensity is 100 mW/cm^2^.

Finally, to testify the application of ZNAs for optoelectronic application, a hybrid solar cell (HSC) was built using sample C with a structure of sample C/P3HT:PCBM/MoO_3_/Ag. Figure 6 shows the typical current density-voltage (*J*-*V*) characteristics of the HSC in the dark and under AM 1.5 illumination with a light intensity of about 100 mW/cm^2^. The device exhibits an open-circuit voltage of 0.51 V, a short-circuit current of 4.53 mA/cm^2^, a fill factor of 39%, and a power conversion efficiency of 0.91%.

## Conclusions

In summary, well-aligned ZNAs were grown on ITO substrates by a facile chemical bath deposition method. Size-selected ZnO nanowires were achieved by changing the precursor concentration, growth temperature, and growth time. The well-aligned ZNAs showed good optical transparency in the visible spectral range, and the nanowires exhibited an excellent crystal quality. A hybrid solar cell composed of the investigated ZNAs with a reasonable power conversion efficiency were realized. This work will be beneficial to develop large-area, low-cost, and high-quality ZNA-based optoelectronic devices in the near future.

## Competing interests

The authors declare that they have no competing interests.

## Authors’ contributions

JSY is the primary author and participated in designing the experiments, experiment analysis and interpretation of data, and language modification. ZLY carried out the experiments, characterization, and acquisition of data. SJH and ZM are the investigators who helped in the analysis and interpretation of data, drafting of the manuscript, and revisions. All authors read and approved the final manuscript.
